# Motivos de no vacunación en menores de cinco años en cuatro ciudades colombianas

**DOI:** 10.26633/RPSP.2017.123

**Published:** 2017-12-20

**Authors:** Fabio Escobar-Díaz, May Bibiana Osorio-Merchán, Fernando De la Hoz-Restrepo

**Affiliations:** 1 Grupo de Epidemiología y Evaluación en Salud Pública Universidad Nacional de Colombia Colombia Grupo de Epidemiología y Evaluación en Salud Pública, Universidad Nacional de Colombia, Colombia.

**Keywords:** Vacunación, esquemas de inmunización, programas de inmunización, cobertura de vacunación, investigación cualitativa, Colombia, Vaccination, immunization schedule, immunization programs, immunization coverage, qualitative research, Colombia, Vacinação, esquemas de imunização, programas de imunização, cobertura vacinal, pesquisa qualitativa, Colômbia

## Abstract

**Objetivo.:**

Conocer las barreras y los motivos de no vacunación en niños y niñas menores de cinco años en algunas ciudades de Colombia.

**Métodos.:**

Diseño cualitativo basado en entrevistas y grupos focales a personal de salud y cuidadores en cuatro ciudades colombianas seleccionadas de acuerdo a diferentes coberturas de vacunación y densidades poblacionales

**Resultados.:**

Se identificaron diferentes factores que pueden influir en el incumplimiento de los esquemas de vacunación en los dos municipios con baja cobertura, como el temor a la reacción posvacunal, las condiciones socioeconómicas, geográficas y de seguridad de la población, las condiciones laborales del personal de vacunación, los problemas administrativos y económicos y el desarrollo precario de los sistemas de información.

**Conclusiones.:**

Desde el punto de vista cualitativo, los equipos de vacunación y los cuidadores destacaron aspectos sociales e institucionales que contribuyen o limitan el cumplimiento de las coberturas de vacunación en las ciudades que participaron en el estudio.

## Abstract (en)

**Objective.:**

Identify barriers and reasons why children under 5 do not get vaccinated in some Colombian cities.

**Methods.:**

Qualitative design based on interviews and focus groups of health workers and caregivers in four Colombian cities selected according vaccination coverage and population densities.

**Results.:**

Different factors were identified that may hinder completion of vaccination schedules in the two municipalities with low coverage, such as fear of a post-vaccination reaction, socioeconomic and geographical conditions and population safety, working conditions of the vaccination staff, administrative and economic problems, and precarious information systems.

**Conclusions.:**

From a qualitative standpoint, the vaccination teams and caregivers pointed to social and institutional issues that may hinder vaccination coverage in the cities that participated in the study.

## RESUMO

**Objetivo.:**

Conhecer as barreiras e os motivos por que não são vacinadas crianças menores de cinco anos em algumas cidades da Colômbia.

**Métodos.:**

Estudo de delineamento qualitativo baseado em entrevistas e grupos focais conduzido com profissionais da saúde e cuidadores em quatro cidades colombianas selecionadas segundo a cobertura de vacinação e a densidade populacional.

**Resultados.:**

Foram identificados diversos fatores para não serem seguidos os esquemas de vacinação nos dois municípios com baixa cobertura: receio de reação pós-vacinal, condições socioeconômicas, geográficas e de segurança da população, condições laborais do pessoal encarregado pela vacinação, problemas administrativos e econô- micos e situação precária dos sistemas de informação.

**Conclusão.:**

Do ponto de vista qualitativo, as equipes de vacinação e os cuidadores destacaram aspectos sociais e institucionais que contribuem para, ou limitam, o cumprimento das metas de cobertura de vacinação nas cidades participantes do estudo.

La vacunación en menores de cinco años es una de las intervenciones más eficaces y costo-efectivas que existen para reducir la mortalidad infantil en el mundo. Se estima que se evitan aproximadamente 2,5 millones de muertes cada año gracias al cumplimiento de un esquema básico de vacunación en niños y niñas. Sin embargo, al menos 20% de los niños que nacen cada año no reciben los beneficios de la vacunación y quedan expuestos a los riesgos de enfermar y morir antes de los cinco años de vida (1).

El Plan Ampliado de Inmunizaciones (PAI) expresa el interés político por lograr coberturas universales en vacunación y, de esta manera, evitar la aparición de enfermedades prevenibles (2). El PAI fue establecido en Colombia a fines de los años setenta y con su implementación se ha logrado, en las últimas décadas, un importante aumento de la cobertura de vacunación que contribuye en la reducción de la mortalidad infantil. Desafortunadamente, las metas de vacunación sufrieron un importante descenso entre los años 2010 y 2011: la cobertura no alcanzó 90% cuando se pretendía que llegara a 95% (3).

Una revisión de estudios publicados entre 1950 y 1990 en todo el mundo sobre motivos de no vacunación (4) encontró que, en los países desarrollados, predominaron aproximaciones con metodologías cuantitativas que excluyeron los aspectos culturales de las comunidades. En contraste, en los países de medianos y bajos ingresos predominaron las investigaciones etnográficas. Los autores concluyen, con esta revisión, que esas investigaciones se han centrado en que los motivos de no vacunación recaen sobre todo en la población como sus creencias o falta de información sobre las vacunas y minimizan el papel de las instituciones y el personal de salud (4).

En los estudios sobre poblaciones con características particulares, como los indígenas y comunidades religiosas, se han empleado principalmente metodologías cualitativas (5–7). En las investigaciones sobre servicios de salud, se han analizado, por medio de encuestas, variables que explican las razones y los motivos de no vacunación como las falsas creencias, la falta de información, el temor a reacciones adversas, las experiencias previas desfavorables, la posición religiosa, la escasa importancia dada por los padres a los efectos de las enfermedades y la desconfianza en las instituciones públicas, entre otros (8–11). En Colombia, los pocos estudios realizados son de carácter transversal y cuantitativos. Sin embargo, sus hallazgos son similares a los que se encuentran en la literatura internacional disponible (12, 13).

Es importante continuar avanzando en la comprensión de los factores o determinantes que han impedido alcanzar las metas esperadas de coberturas de vacunación. Es indispensable entender el papel de los servicios de vacunación, los recursos humanos, técnicos y administrativos en el problema de la baja cobertura. Así, el presente estudio tuvo como objetivo principal identificar los motivos y las barreras que influyen en el incumplimiento de los esquemas de vacunación en niños y niñas menores de cinco años en cuatro ciudades colombianas, desde la perspectiva de los funcionarios responsables del PAI en estas entidades territoriales y, también, con la opinión de algunos cuidadores.

En 1994 comenzó la implementación del Sistema General de Seguridad Social en Salud (SGSSS) bajo un modelo de aseguramiento universal que posee un régimen contributivo y uno subsidiado de acuerdo a la capacidad de pago de las personas. Estas deben afiliarse a una empresa promotora de salud (EPS) y, por medio de una institución prestadora de servicios de salud (IPS), reciben un paquete de beneficios en salud. Las vacunas incluidas en el PAI forman parte de estas prestaciones, son gratuitas y las autoridades del sector y las instituciones públicas y privadas en el sistema de salud tienen la responsabilidad de garantizar estos servicios (14, 15).

## MATERIALES Y MÉTODOS

El diseño metodológico cualitativo tuvo como intención comprender la vacunación como un fenómeno social, en el cual existen significados y subjetividades alrededor de ella (16). Se realizaron entrevistas individuales y grupos focales, estrategias con las cuales se analizó el lenguaje para aproximarse a la realidad vivida y sentida por las personas (17). Se diseñó y aplicó una guía de entrevista flexible con temáticas relacionadas con información individual, demográfica y laboral, organización del servicio de vacunación en el municipio, fortalezas y debilidades del programa y razones que impiden cumplir con las coberturas.

La selección de las cuatro ciudades se realizó de acuerdo a su densidad poblacional y las coberturas de vacunación, las cuales podían ser altas o bajas en cada una de ellas. Esta escogencia, por tanto, no obedeció a un criterio de representatividad estadística, sino de comparabilidad entre estas ciudades de acuerdo a estos criterios (cuadro 1). Cada ciudad está ubicada en una región de Colombia: Soacha en el centro del país, Leticia en la región amazónica, Barranquilla en la costa atlántica y Quibdó en la costa pacífica.

En las entrevistas y grupos focales participaron los coordinadores del PAI y los equipos de vacunadores de las instituciones de salud públicas o privadas, y de las autoridades públicas en salud en las ciudades consideradas para el estudio. De manera complementaria, se hicieron estas mismas estrategias para conocer la experiencia de padres, madres y cuidadores presentes en los servicios de vacunación, con el fin de conocer las dificultades y razones que han impedido cumplir con el esquema de vacunación de los niños y niñas.

Se empleó el criterio de conveniencia para la selección de la muestra cualitativa de acuerdo al tipo de informante, como coordinadores y vacunadores, y entidades públicas o privadas Por último, se aplicó el criterio de redundancia o saturación de datos cuando la información era repetitiva y, de este modo, se detuvo la realización de más entrevistas (18).

Se realizaron en total 36 entrevistas individuales y seis grupos focales, que fueron registrados mediante una grabadora digital de audio, con el consentimiento de los participantes. Todos eran mayores de edad y manifestaron verbalmente su disposición a participar en el estudio. Se les comunicó con claridad los objetivos de la investigación y se les garantizó la confidencialidad de los resultados.

El análisis se realizó por medio de la lectura de las transcripciones, codificación, visualización, reducción e interpretación (19, 20). Para garantizar el rigor metodológico en este tipo de investigaciones, se usaron los criterios de credibilidad (transcripciones textuales como fuente primaria, triangulación con otros estudios), auditabilidad (uso de grabadora, descripción de los informantes y transcripción textual) y transferibilidad (descripción de la codificación) (17). El esquema de categorías es el siguiente:

**CUADRO 1.** Características demográficas y de cobertura vacunal en cuatro ciudades colombianas

Ciudad	Densidad poblacional (habitantes/km^2^)	Cobertura de vacunación (%)	VOP	DPT	BCG	HB	HiB	RT
Barranquilla	Alta (700)	Alta	97,6	98,7	116,1	98,7	98,7	84,7
Soacha	Alta (286)	Baja	69,1	69,1	42,1	69,3	69,1	48,3
Leticia	Baja (5,6)	Alta	98,3	98,3	109,5	98,3	98,3	86,2
Quibdó	Baja (3,4)	Baja	87,1	87,1	120,6	87,1	87,1	72,7

***Fuentes:*** Ministerio de Salud y Protección Social y Departamento Nacional de Planeación, Colombia.

VOP, vacuna oral para la poliomielitis; DPT, vacuna triple bacteriana (difteria, tétanos y tos convulsa); BCG, vacuna para la tuberculosis; HB, vacuna para la hepatitis B; HiB, vacuna para el *Haemophilus influenzae*; RT, vacuna para el rotavirus.

1. Información, creencias y actitudes de la población
  - Temor a la reacción posvacunal
  - Aspectos culturales
2. Condiciones socioeconómicas y geográficas
  - Seguridad y orden público
  - Transporte y vías de comunicación
  - Migración
3. Situación institucional de los servicios de vacunación
  - Condiciones de trabajo de los equipos de vacunación
  - Limitaciones administrativas
  - Sistemas de información a nivel local
  - Papel de aseguradores y prestadores privados
  - Rivalidad entre prestadores por metas en vacunación

## RESULTADOS

### Información, actitudes y creencias sobre la vacunación

Para algunos cuidadores, el temor a la reacción posvacunal, como el dolor y la fiebre, es una de las razones por las cuales no se vacunan a los niños menores de cinco años: “*cuando yo vacuné a mi hija, la vacuna de los dos meses se la coloqué en este servicio de salud y ella duró quince días enferma con fiebre, vómito y diarrea*”. Frente a esto, un coordinador del PAI manifestó: “*Son muchas las familias que siguen con la concepción de que dejan perder las oportunidades de vacunación o no asisten a sus citas porque los niños se enferman, es decir, todavía tienen esa idea de que la vacuna los enferma, de que van a llorar mucho*

Aunque el PAI es gratuito para toda la población dentro del territorio nacional, algunas familias se abstienen de vacunar a sus hijos menores de cinco años cuando, por ejemplo, alguno de los padres pierde su empleo y, aunque la atención en salud se suspende temporalmente, desconocen que el servicio de vacunación sí se mantiene. Un integrante del equipo de vacunación municipal afirmó que: “… *hay niños que han estado atrasaditos en las vacunas, que pasan del año y tienen el esquema atrasado porque los padres creen que como ya no tienen afiliación a la EPS, entonces ya no los van a atender en vacunación*”. Algo similar indicó el vacunador de uno de los centros de salud:*“… si no tienen ningún tipo de aseguramiento creen que en ningún lado se pueden vacunar porque no tienen carnet de SISBEN [Sistema de Identificación de Beneficiarios]”*.

El temor a la reacción posvacunal fue una categoría presente en las cuatro ciudades, resaltado también por los equipos locales de vacunación. Sin embargo, fue más frecuente en aquellas donde las coberturas eran bajas, como Quibdó y Soacha.

### Condiciones socioeconómicas y geográficas

De acuerdo al personal de vacunación, el conflicto armado, la delincuencia y la distancia geográfica se convierten en una barrera para el acceso a estos servicios y, por lo tanto inciden, en la decisión de no vacunar, o no hacerlo en forma oportuna. En Quibdó, según la coordinadora PAI: “*aquí ya tenemos zonas calientes, por ejemplo la zona norte, es una zona donde la mayoría de población es desplazada, y eso allí ya hay problemas de orden público, la jornada última del año pasado no pudimos ir en el tiempo que era porque había un paro armado ( ) es muy difícil que yo envíe un grupo de vacunadores que los están esperando para robarlos, esa parte de seguridad es la que de pronto afecta el proceso de vacunación*”.

En Quibdó hay dificultades para la movilización de las personas que habitan zonas lejanas debido a la falta de transporte fluvial permanente, el bajo nivel del río durante el verano, que hace difícil su navegación, y las vías terrestres que no son transitables durante el invierno. Estas circunstancias impiden que los niños puedan desplazarse hasta el centro de vacunación o que los vacunadores logren llegar hasta su sitio de residencia, quienes no cuentan con vehículo disponible para esta actividad: *“para el área carreteable no contamos con un vehículo y lo mismo aquí en la zona urbana no contamos con un vehículo: para las jornadas se contrata un vehículo, que lleve las vacunadoras, que las deje por allá donde corresponde, y luego las traiga de regreso”*.

Además, el desplazamiento de las familias por la violencia o por motivos económicos dificulta que los niños y niñas cumplan con su esquema de vacunación. La coordinadora PAI de la ciudad de Quibdó manifestó que los niños *“se desafilian, porque se han ido a Medellín, que viven en otro lugar, se retiran y no avisan. Porque eso yo lo noto a cada rato, reviso que me faltan muchos niños y me dicen ‘no, ya nos trasladamos para otro lugar’ ”*.

### Condiciones institucionales y de los servicios de salud

Dentro de las instituciones de salud, se identificaron problemáticas que pueden incidir en las coberturas de vacunación de los municipios. Así, en Quibdó y Soacha, cuyas coberturas son bajas, las personas responsables del PAI tienen muchas funciones a cargo además de las que son propias del programa. Por otra parte, los equipos de vacunación sufren de una alta rotación y tienen bajos ingresos, y en algunas ocasiones no cuentan con los implementos adecuados para la prestación de este servicio. Una coordinadora del PAI expresó que: *“la auxiliar aunque está capacitada para vacunación tiene otras funciones, es decir no es exclusiva del PAI, ella tiene que ayudarme a mí, atender al médico, al especialista”;* otra coordinadora afirmaba: *“[yo] contrato el grupo de vacunadores, pero no les doy la continuidad y esto se va a nivel municipal y de las EPS públicas y privadas, resulta que la señora tiene contrato con una cooperativa en la institución y la tiene por dos años y luego la cambia”*.

A lo anterior, se agregan los demorados trámites para la contratación de los equipos de vacunación municipal, debido a la tardía aprobación de los recursos económicos destinados para este propósito, como sostiene la coordinadora del PAI de Soacha: *“contratan el grupo extramural en diciembre y lo contratan por tres meses, solo hasta marzo, y ahí ya queda una brecha porque desde noviembre hacia atrás yo no hice la búsqueda activa de esos niños, esa estrategia no fue efectiva, si no puedo tener el personal y los recursos; se me vuelve una dificultad cuando a nivel administrativo se está retrasando ese proceso. Entonces no alcanzo, así yo tenga el recurso, el personal en esas condiciones, yo no los puedo quemar y en un mes pedirles que hagan lo de todo un año*”.

Otro elemento que se resaltó especialmente en los municipios con bajas coberturas es la carencia de un sistema de información en vacunación. La coordinadora del PAI de Quibdó manifestó que: *“es una desventaja no tener un sistema de información único a nivel municipal, es muy dispendioso para una vacunadora buscar por ejemplo un niño que se vacunó en el 2008, hay y empezar a buscar en los registros del 2008 en las sábanas [registro en papel]. A nivel institucional tenemos todavía esa cultura, hay archivos desde el 2005, 2006, 2008 hasta la fecha, y todo eso está en carpeticas, está guardado. Es dispendioso porque nos toca desgastarnos buscando en los archivos, mientras divinamente lo podríamos hacer por el sistema*”. Esta situación genera problemas de seguimiento oportuno y preciso a las coberturas de vacunación de los niños y niñas de estas ciudades.

Por otra parte, según las coordinadoras del PAI de Soacha y de Quibdó, como el programa es gratuito, público y está incluido en las prestaciones individuales y colectivas del SGSSS, las entidades aseguradoras no hacen el suficiente esfuerzo para hacer un seguimiento sus afiliados y cumplir sus metas de vacunación, sino que delegan esta función a las instituciones públicas municipales de salud. Así, una de ellas comentaba que: *“Se supone que año a año se nos viene diciendo a los entes territoriales que las coberturas de vacunación son responsabilidad de las EPS toda vez que esto está dentro del POS (…) entonces la coordinadora PAI tiene ese 95% de cobertura en el hombro y Caprecom, Selvasalud o cualquiera de las EPS contributivas o subsidiadas duermen todos los días y no tienen que contestar a la Supersalud, absolutamente nada”*.

En Soacha, algunos cuidadores manifestaron que la aplicación de las vacunas en el centro de salud se ha visto afectada por la cantidad de niños o niñas que lleguen él y así poder usar las dosis disponibles: *“varias veces traje a mi hija al servicio para la vacunación y me dijeron que hasta que no hayan catorce niños no se pueden vacunar, porque no pueden sacar las dosis”*. En otros casos, cuando se excedía el número de niños durante el día y se requerían mas dosis, quien requiriera el servicio debía ir otro día: “… *yo lleve mis dos niños a vacunar y me la programaron las vacunas para una fecha, no pude el mismo día, dijeron que hoy no se podía porque ya están completas las 12 vacunas para hoy, y así me tienen”*.

En este municipio, su cercanía geográfica con Bogotá D.C., capital de Colombia, es considerada como un factor que incide en las bajas coberturas, según la coordinadora PAI: “*a mí me nacen al mes entre 200 y 250 niños, mensualmente en la única IPS que presta el único servicio de sala de partos, que es el Hospital Mario Gaitán; sin embargo, dentro de mi reporte hay más población, y esos niños qué pasa, son mamitas de alto riesgo que son remitidas a que sus niños nazcan en Bogotá, y esa población es vacunada allá, pero tampoco es reportada para mí, entonces mi cobertura de BCG [vacuna para la tuberculosis] siempre permanece baja, rojita, porque mi población está allá”*. De acuerdo con esta situación, una parte de la población de Soacha reciben la atención en salud en las instituciones de Bogotá D.C; la vacunación es una de ellas, por tanto la información queda registrada para la capital contribuyendo a sus propias metas y el municipio pierde ese dato.

## DISCUSIÓN

Este estudio identificó diferentes barreras y motivos de no vacunación en niños y niñas menores de cinco años en cuatro ciudades colombianas. Se compararon según dos características fundamentales: su densidad poblacional y su cobertura de vacunación. Así, hay dos municipios con coberturas bajas (Soacha y Quibdó) y dos con coberturas altas (Barranquilla y Leticia). Soacha tiene una alta densidad poblacional mientras que en Quibdó es baja y cuenta con una importante población que está dispersa. En todas las ciudades, aun se destacan los temores y las creencias erróneas sobre las vacunas y el desconocimiento sobre la gratuidad del servicio de vacunación.

Hay algunos elementos que parecen ser más predominantes en los dos municipios con menor cobertura, a pesar de tener densidades poblaciones diferentes y que provienen de su contexto social e institucional. Soacha y Quibdó comparten dificultades comunes como la inseguridad o el conflicto armado, que restringen el acceso a los servicios de vacunación. Además, el desplazamiento forzado por la violencia o simplemente por motivos económicos también pueden influir en la escasa cobertura y a los esquemas incompletos de vacunación. Para el caso de Quibdó, sus caracteristicas geográficas y la ubicación dispersa de parte de su población son factores adicionales junto con la falta de medios de transporte fluvial y terrestre.

Con respecto al aspecto institucional, en los municipios con bajas coberturas suelen presentarse dificultades administrativas que demoran la oportuna contratación del personal de vacunación; además, hay multiplicidad de funciones, inestabilidad laboral, alta rotación y, en algunos casos, no hay vehículo ni implementos necesarios para el cumplimiento de las actividades propias del PAI. Por otro lado, algunas entidades aseguradoras presentes en estos municipios carecen de compromiso en garantizar los esquemas de vacunación de sus poblaciones afiliadas y delegan esta responsabilidad en el municipio. Por último, los sistemas de información local no han sido desarrollados en forma adecuada, lo cual hace dificil llegar a un registro y seguimiento confiable sobre sus coberturas de vacunación.

Se destaca en este estudio que no surgieron motivos relacionados con las creencias tradicionales que pudieran influir en las bajas coberturas, resultado diferente a lo manifestado en otras investigaciones como la que fue realizada en una comunidad mexicana para conocer las causas de no vacunación (6), la que identificó los determinantes de la reticencia de los padres hacia la vacunación en una ciudad africana (7) o en aquella que consideró las percepciones sobre la vacunación infantil en una comunidad europea (5).

Por lo tanto, los resultados indican que hay motivos que provienen tanto de las personas y su contexto social y geográfico como de las instituciones de salud. Esta interpretación coincide con un estudio realizado en Brasil, donde se encontró que tanto la resistencia a la vacunación como la falta de insumos restringen el cumplimiento de las metas esperadas (21).

Algunos estudios realizados en Colombia han analizado cómo el funcionamiento del SGSSS y los procesos de descentralización en salud a nivel local han afectado la adecuada respuesta institucional a algunos eventos de interés en salud pública (22–25). Con respecto a la inmunización, una investigación analizó el PAI en tres municipios colombianos en relación con el SGSSS y encontró que estos no estaban preparados para los profundos cambios institucionales que implicó el nuevo sistema de salud desde 1994. Esto se tradujo en la reducción de coberturas de vacunación entre los años 1995 y 2000 (26).

La limitación más importante de este estudio fue la imposibilidad de acceder a las familias o cuidadores con niños y niñas que tuvieran esquemas de vacunación incompletos en el momento de la recolección de información. Dados los resultados encontrados, es importante profundizar los análisis tanto cualitativos como cuantitativos en otras ciudades con bajas coberturas en vacunación para comprender mejor cuáles son las barreras, situaciones y motivos que inciden en el incumplimiento de los esquemas de vacunación de los menores de cinco años, considerando por tanto los aspectos sociales, culturales, geográficos, económicos e institucionales, aspectos que aún siguen siendo poco entendidos en el país y que cuyo conocimiento podría contribuir a ajustar y fortalecer el PAI de acuerdo a estos elementos en el nivel nacional, regional y local.

De este modo, el estudio brindó importantes elementos de carácter cualitativo para avanzar en la comprensión de los motivos y barreras percibidas por los equipos de vacunación y por los cuidadores que determinan el cumplimiento o no de las coberturas de vacunación en ciudades con diferentes densidades poblacionales y que se ubican en diferentes regiones del país.

### Financiamiento

Este documento hace parte del estudio realizado por el Grupo de Epidemiología y Evaluación en Salud Pública, Universidad Nacional de Colombia, financiado por el Ministerio de Salud y Protección Social, mediante convenio interadministrativo desarrollado en el año 2012.

### Declaración

Las opiniones expresadas en este manuscrito son responsabilidad del autor y no reflejan necesariamente los criterios ni la política de la RPSP/PA-JPH y/o de la OPS
